# Changes in Photosynthesis Could Provide Important Insight into the Interaction between Wheat and Fungal Pathogens

**DOI:** 10.3390/ijms22168865

**Published:** 2021-08-18

**Authors:** Huai Yang, Peigao Luo

**Affiliations:** Provincial Key Laboratory for Plant Genetics and Breeding, College of Agronomy, Sichuan Agricultural University, Chengdu 611130, China; yanghuai202103@163.com

**Keywords:** photosynthesis, wheat diseases, immune defense, ROS, gene expression

## Abstract

Photosynthesis is a universal process for plant survival, and immune defense is also a key process in adapting to the growth environment. Various studies have indicated that these two processes are interconnected in a complex network. Photosynthesis can influence signaling pathways and provide both materials and energy for immune defense, while the immune defense process can also have feedback effects on photosynthesis. Pathogen infection inevitably leads to changes in photosynthesis parameters, including Pn, Gs, and Ci; biochemical materials such as SOD and CAT; signaling molecules such as H_2_O_2_ and hormones; and the expression of genes involved in photosynthesis. Some researchers have found that changes in photosynthesis activity are related to the resistance level of the host, the duration after infection, and the infection position (photosynthetic source or sink). Interactions between wheat and the main fungal pathogens, such as *Puccinia striiformis*, *Blumeria graminis*, and *Fusarium graminearum*, constitute an ideal study system to elucidate the relationship between changes in host photosynthesis and resistance levels, based on the accessibility of methods for artificially controlling infection and detecting changes in photosynthesis, the presence of multiple pathogens infecting different positions, and the abundance of host materials with various resistance levels. This review is written only from the perspective of plant pathologists, and after providing an overview of the available data, we generally found that changes in photosynthesis in the early stage of pathogen infection could be a causal factor influencing acquired resistance, while those in the late stage could be the result of resistance formation.

## 1. Introduction

Photosynthesis is a universal process in the plant kingdom that occurs in various green organs, such as leaves [1], young stems [2], green fruits [3], and ears before maturity [4], providing a material basis and energy supply for multiple physiological metabolic processes in plants [5]. Plant organs that can perform photosynthesis are considered photosynthetic source organs, which mainly include the leaves of plants, while the storage organs of the organic matter synthesized by photosynthesis represent photosynthetic sink organs, which include mainly stalks, roots, and fruits [6]. At different growth and development stages, the photosynthetic sources and sinks can change accordingly. For instance, when plants are in the seedling stage, leaves and stems act as both sources and sinks of photosynthesis because photosynthates are supplied for their own growth and development. Photosynthesis is disrupted by a variety of complex factors, including abiotic stresses caused by water [7], temperature [8], light [9], and mechanical damage [10] and biological stresses caused by insects and pathogens [11,12]. In contrast, photosynthetic changes during this process may be related to responses to these factors.

Photosynthesis is sensitive to abiotic stress. Lack of water can limit photosynthesis efficiency due to thylakoid membrane damage and reduced chlorophyll contents [13]. Both high and low temperatures inhibit the activities of photosynthesis-related enzymes and membrane-associated electron carriers, thus further reducing the rate of photosynthesis [14,15]. Light intensities higher than the light saturation point of photosynthesis can cause photoinhibition of photosystem II (PSII) [9], and limited irradiance also directly leads to a decline in the photosynthesis rate [16]. In addition, complex photosynthetic changes can also be caused by the invasion of various insects and pathogens. For example, the photosynthesis rate of rice leaves is inhibited by *Nilaparvata lugens* infestation, especially in the lower part of the leaves [17], and the net photosynthesis rate (Pn) and related parameters of susceptible tomato leaves are affected by inoculation with *Fusarium oxysporum* f. sp. *lycopersici* or *Verticillium albo-atrum* [18]. The photosynthesis capacity of maize leaves is also inhibited by infection with *Colletotrichum musae* and *Fusarium moniliforme*, and the inhibition is accompanied by a sharp decrease in chlorophyll content [19]. Changes in photosynthesis in response to changes in environmental conditions constitute a traditional and interesting topic for scientists in the field of photosynthesis. However, the objective of this review is to underscore photosynthetic changes during pathogenesis from the standpoint of pathologists.

It is well known that most pathogen invasions lead to a decline in the photosynthesis rate of the host [20,21]. However, various authors carefully monitored the photosynthetic changes during pathogen infection in a host and detected a very significant phenomenon: in the early stages of infection by a large number of different pathogens, the photosynthesis capacity of the host always decreases [22,23,24,25]. For example, photosynthesis in both resistant and susceptible barley decreased after inoculation with powdery mildew, and moreover, the decrease in photosynthesis in the resistant plants was larger than that in the susceptible plants [22]. Similar decreases were also observed in interactions between tobacco and *Phytophthora nicotianae* [23] and between *Arabidopsis* and *Pseudomonas syringae* [26]. These findings indicate that the difference in the degree of photosynthetic changes in the early stages of infection could conversely be an indicator of the resistance level.

## 2. Wheat and Its Fungal Pathogens Constitute Ideal Pathogen Systems for Studying the Role of Photosynthesis in the Process of Resistance Development

### 2.1. Characteristics of the Three Major Fungal Diseases of Wheat

Wheat is one of the most important crop species and has been a staple food of humans for thousands of years worldwide [27]. The growth period of wheat is longer than that of other food crop species such as maize and rice, making wheat more susceptible to various pathogens and diseases during its growth and development [28]. Among these diseases, the wheat powdery mildew caused by the obligate biotrophic fungus *Blumeria graminis* f. sp. *tritici* (*Bgt*), stripe rust caused by the biotrophic parasite *Puccinia striiformis* f. sp. *tritici* (*Pst*), and Fusarium head blight (FHB), which is caused mainly by *Fusarium graminearum* species, are the three most severe diseases affecting both yield and quality [29,30]. The basic characteristics of the three diseases are listed in Table 1. Wheat powdery mildew is an epidemic disease worldwide, especially in regions such as southwestern China, whose environment is temperate and rainy during the wheat growing season [31]. Furthermore, powdery mildew can occur throughout the whole growth period of wheat, and the optimal temperature for its occurrence is 15–20 °C [32]. For the infected position, *Bgt* pathogens infect mainly the leaves and sometimes also infect green awns, glumes, and stalks [33]. These organs differ largely in the position of the plant, but they are usually considered photosynthetic source organs at the developmental stage because of their strong photosynthetic competence [34]. Therefore, wheat powdery mildew is a typical source disease from the perspective of photosynthetic function. Wheat stripe rust is also one of the most threatening diseases in wheat production worldwide [35], especially in temperate climates. Stripe rust can occur during the whole growth period from seedling emergence to maturity, and the optimum temperature for its occurrence is 13–16 °C [36,37]. The tissue specificity of stripe rust is stronger than that of wheat powdery mildew, which mainly parasitizes leaves, the main source of wheat photosynthesis, and seldom occurs in wheat stems and glumes [38]. Therefore, wheat stripe rust is also a typical source disease from the perspective of source–sink relationships.

It is well known that the occurrence of FHB in wheat is extremely harmful to the quality of wheat because the causal agent produces the mycotoxin deoxynivalenol (DON), which is poisonous to both humans and animals [40]. At the same time, FHB can also cause great loss of wheat yield, especially under the environment conditions suitable for *F. graminearum* growth and breeding [41,42]. *F. graminearum* infects mainly the ears of wheat at the adult stage, and they are generally considered sink organs due to their role as a storage location for photosynthates in wheat. Therefore, in contrast to wheat powdery mildew and stripe rust, FHB is a typical sink disease. Measurements of photosynthesis parameters of FHB-resistant/susceptible sister-line wheat after inoculation with *F. graminearum* showed that the photosynthesis rate of susceptible wheat leaves did not change significantly, while the photosynthesis rate of resistant wheat leaves decreased significantly in the early stage after pathogen infection, but the resistant genotype produced a larger yield than the susceptible genotypes did [43]. This indicates that the decrease in photosynthesis rate could play a key role in inducing systemic resistance to maintain the ultimate yield.

### 2.2. Wheat and Its Tissue-Specific Diseases Constitute an Ideal System

The mechanism through which both stripe rust and powdery mildew cause yield losses could be different from that of FHB because of the difference in the infection positions. A variety of wheat leaf diseases can reduce the amount of green leaf area, resulting in a decrease in chlorophyll contents in the infected parts [44], which directly reduces the photosynthesis capacity and both the synthesis and the accumulation of organic matter in photosynthetic source organs, leading to a decline in wheat yield [45]. For instance, the chloroplast envelope of wheat mesophyll cells is disrupted after infection with *Bgt*, and the thylakoid becomes enlarged [45]. The effector protein of wheat stripe rust fungus can inhibit chloroplast function [46], and *Pst* infection can also reduce the chlorophyll content in wheat leaves [24]. However, there may be several reasons for wheat yield loss caused by FHB. Studies have indicated that damaged photosynthetic sink organs may inhibit photosynthesis at the source via feedback mechanisms, which could be the important reason to explain the yield loss caused by FHB [43,47].

In addition, a few reports have indicated that photosynthesis may be involved in wheat immune defense responses to fungal pathogens and may influence resistance formation because changes in photosynthesis may provide an important signal for maintaining source—sink balance during interactions between wheat and pathogens [12,48]. However, there are few relevant research reports on the role of photosynthesis in influencing the formation of wheat resistance, and those studies have focused mainly on photosynthetic changes after inoculation with pathogens, so knowledge about the influence of photosynthesis is very fragmented or not systematic [49]. Both wheat powdery mildew and stripe rust are source organ diseases, and FHB is a sink organ disease. Hence, wheat and its fungal pathogens constitute an ideal pathogen system for exploring the role of photosynthesis in the development of wheat resistance.

## 3. Relationships between Yield and Photosynthetic Changes Caused by Pathogen Infection in Wheat

### 3.1. Effects of Photosynthetic Changes on Wheat Yield

Decreases in wheat yield caused by pathogen infection have always been a focus of wheat breeders and physiologists [50]. A large number of studies have reported adverse effects of wheat leaf diseases (such as powdery mildew and stripe rust) and ear diseases (such as FHB) on wheat yield [51,52]. Infection of wheat powdery mildew at the seedling stage can affect the growth and development of wheat plants [53] and can further lead to a decrease in grain filling and grain weight at the adult stage [33]. Wheat stripe rust infection not only can reduce the number of tillers at the tillering stage [54] but also can significantly reduce the grain number per spike and 1000-grain weight at the adult stage [55]. Wheat stripe rust and powdery mildew have very similar effects on wheat yield because they both directly act on the source organs of wheat photosynthesis and cause wheat yield losses through long-term inhibition of photosynthesis in wheat leaves [52,56,57]. In addition, both *Bgt* and *Pst* are parasitic fungi that rely on host metabolism to provide carbohydrates, amino acids, and inorganic nutrients [58,59]. The growth and reproduction of a large number of pathogens directly consume nutrients in photosynthetic source organs, further reducing wheat yields [58].

The production of the mycotoxin DON in diseased wheat ears is the main negative effect caused by FHB but could also lead to severe yield losses due to a damaged photosynthetic sink and a disrupted source–sink balance [40,43]. It has been reported that FHB can cause 10–70% yield losses in years in which epidemics occur [45]. Unfortunately, how the damage of photosynthetic sink organs causes the changes of photosynthetic source organs remains to be further explored. It has been reported that the activity of photosynthesis-related enzymes and the expression of associated gene transcripts are modified by sink demand [60]. Stomatal closure is a plant’s first line of defense against pathogens [61]. Recent studies have suggested that the reduced photosynthetic efficiency of susceptible wheat leaves is regulated by stomatal factors after the occurrence of FHB symptoms, while the Gs of resistant wheat leaves did not show a significant decrease under the same treatment [43,50]. These reports suggest that when pathogens attack the photosynthetic sinks of wheat, there is some kind of feedback regulatory mechanism that alters the balance between photosynthetic sources and sinks by adjusting photosynthesis parameters.

### 3.2. Association between Changes in Photosynthesis and Immune Defense in the Early Stages after Pathogen Infection

In the past, plant defense responses and photosynthesis have been studied independently. With the mechanisms underlying plant photosynthesis and immune defense gradually becoming clear, it has been found that plant photosynthesis can provide materials and can serve as a signal transduction basis for plant immune defense [49,62], which indicates that plant photosynthesis and immune defense processes are interconnected [63]. A series of reports have shown that host photosynthesis usually declines in the early stages of invasion by various pathogens [25,64,65,66], which may be involved in the immune response of the host. For instance, in incompatible reactions between tobacco and *P. nicotianae*, the photosynthetic electron transport chain in tobacco leaves is disrupted, and photosynthetic activity decreases with the accumulation of reactive oxygen species (ROS) just a few hours after inoculation [23]. Moreover, when interactions between *Arabidopsis* and *P. syringae* strains were analyzed, it was found that both virulent and avirulent strains of *P. syringae* could cause a decrease in photosynthesis in *Arabidopsis* leaves, and a decrease in photosynthesis was detected within only 3 h after pathogen inoculation during incompatible reactions [24]. Although the molecular mechanism underlying the decrease in photosynthesis caused by pathogen infection in the early stage remains unclear, recent studies on photosynthetic changes caused by various pathogens have provided several clues to help elucidate the role of photosynthetic changes in the defense response.

Infection of wheat pathogenic fungi in the leaves and ears can cause a decrease in photosynthesis in wheat leaves. The decline in photosynthesis in the early stage of various fungal pathogens invasion in wheat are listed in Table 2. In incompatible reactions, the decrease in photosynthesis capacity of the host may be due to the material and energy needed for defense [67], while in compatible reactions, the decrease in photosynthesis capacity may be due to the damage caused by pathogen infection of the host [68,69]. Unlike the transcriptomic and photosynthetic changes in sister wheat lines after inoculation with *Bgt.*, it was found that the inhibition of photosynthesis in resistant wheat paralleled the global downregulation of photosynthesis-related genes to actively regulate the immune response, but the decrease in photosynthesis in susceptible wheat lines is caused by stomatal closure and did not regulate the immune response [25]. The infection of wheat stripe rust also causes a phenomenon similar to that caused by powdery mildew. The Pn of the resistant cultivar CN19 carrying the gene *Yr41* [70] and the susceptible cultivar Sy95-71 decreased significantly at 72 h after inoculation with *Pst* compared with no inoculation [24]. By exploring the resistance mechanism of wheat resistance genes, it was also found that, by inhibiting photosynthesis, the stripe rust resistance gene *Yr36* could provide broad-spectrum resistance to *Pst* races in wheat at the adult stage [71]. Studies on the signals of photosynthetic changes caused by wheat leaf diseases have also suggested that the resistant genotypes of wheat could actively regulate photosynthetic changes to mediate specific immune defenses against the invasion of pathogens, albeit at a cost of greatly reduced photosynthesis capacity in the initial stage of pathogen infection [72]. Taken together, these results indicate that the changes in photosynthesis parameters in the early stage of stripe rust and powdery mildew infection in wheat were related to the development of resistance.

Interestingly, photosynthesis-related parameters in the ears and leaves of resistant and susceptible wheat were found to be significantly different after inoculation with *F. graminearum* [74]. The photosynthesis of wheat spikes increased at the early stage of inoculation in both resistant and susceptible plants, although the spikes were directly infected by *F. graminearum*; however, there was a larger increase in the ears and a larger decrease in the leaves in the resistant plants than in the susceptible plants [74]. Therefore, changes in the photosynthesis parameters of different genotypes of wheat may provide important insight into the mediation of immune defense responses to wheat sink diseases.

## 4. ROS Produced by Photosynthesis Relay Compatibility or Incompatibility Signals

Photosynthesis can regulate the immune defense response in many ways, the most important of which is by inducing the production of ROS and regulating changes in hormones [12,48,67]. ROS, including singlet oxygen (^1^O^2^), superoxide anion radicals (O^2•−^), hydrogen peroxide (H_2_O_2_), and hydroxyl radicals (•OH) are produced mainly during interactions between metabolic intermediates and oxygen during photosynthesis [75,76]. The concentration of ROS in plants is maintained usually at low levels due to rapid and precise regulation by various antioxidant enzymes, mainly catalase (CAT), superoxide dismutase (SOD), ascorbate peroxidase (APX), and peroxidase (POD) [76]. The metabolism of ROS has a dual role in plant growth and development. On the one hand, high concentration of ROS accumulation in plant cells can ultimately damage proteins, lipids, and nucleic acids and can even disrupt photosynthesis, and ROS are toxic to many cellular processes in plants [77,78,79]. On the other hand, a role for ROS in the immune defense has gradually been elucidated, indicating that ROS play a crucial role in plant immune defense [80]. The production of ROS is one of the earliest cellular reactions following pathogen recognition [81]. Many studies have revealed that low concentrations of ROS can activate the expression of defense-related genes and induce various defense responses [82], while a high accumulation of ROS can be used as a defense weapon to resist pathogen invasion [83]. In addition, ROS can regulate immune defense through interactions with plant hormones [84,85], and the synergistic effect of ROS and salicylic acid (SA) plays an important role in mediating the hypersensitive response (HR) [86,87]. In interactions between plants and pathogens, the ROS that accumulate are generated mainly by photosystem I (PSI) and PSII during photosynthesis [88]. Therefore, the changes in ROS caused by photosynthetic changes could be an important factor explaining why early photosynthesis is involved in the development of plant disease resistance, which ultimately results in compatibility or incompatibility between pathogens and hosts.

A large number of studies have suggested that the accumulation of ROS, particularly H_2_O_2_, constitutes an important signal that is transmitted and may determine the compatibility between pathogenic fungi and wheat in the early stage of infection [69,73,89]. For instance, transgenic experiments confirmed that, after being transferred to a resistance gene, the powdery mildew resistance of susceptible wheat variety Yangmai 158 was significantly improved at the seedling and mature stages and accumulated more ROS at the *Bgt* infection position [90]. The results indicate that ROS could improve the resistance of wheat to powdery mildew. By measuring the changes in ROS and photosynthesis in resistant/susceptible sister wheat lines after inoculation with *Bgt*, Hu et al. [25] reported that two stages of H_2_O_2_ bursts occurred during the incompatible reaction process and that a single low-amplitude and transient H_2_O_2_ outbreak in susceptible wheat lines was not sufficient to induce the HR. In addition, ROS signaling plays a crucial role in the immune response to stripe rust caused by the parasitic fungus *Pst*. Overexpression of LSD-1-like zinc-finger protein (*TaLOL)*, which actively regulates the ROS signaling pathway, can enhance resistance to stripe rust by inducing the production and accumulation of ROS and cell death, while silencing *TaLOL2* increases sensitivity to avirulent races of *Pst* and reduces ROS production in wheat [73]. These results indicate that the accumulation of ROS acts as an important signal of immune defense in wheat leaf diseases caused by parasitic fungi.

FHB resistance is an extremely complex trait that is controlled by multiple quantitative trait loci (QTLs) and includes the additive effect of several genes [91]. Wheat resistance to FHB consists of two main types: resistance to the initial infection (type I resistance) and resistance to the spread of the disease within the wheat head (type II resistance) [92]. *F. graminearum*, the causal agent of wheat FHB, is a hemibiotroph that can absorb nutrients from dead tissues, so the HR mediated by the accumulation of ROS may not easily effectively resist FHB [93]. The results of some studies on the changes in ROS levels during FHB infection are consistent with our views. For instance, comparative transcriptome analysis suggested that ROS may accumulate in susceptible mutants of the wheat cultivar Wangshuibai after inoculation with *F. graminearum* but not in FHB-resistant cultivars of Wangshuibai [94]. Another example is the FHB-resistant line L693, which exhibits temporary infection symptoms due to insufficient accumulation of ROS after inoculation with *F. graminearum*, after which systemic acquired resistance (SAR) is induced in distal tissues via the SA pathway to resist pathogen invasion [74]. These results indicate that type II resistance to spreading within the wheat head mediated by the jasmonic acid (JA) or SA pathway may determine the compatibility between pathogens and wheat instead of mediating a strong immune defense response based on ROS at the site of parasitic fungal infection [74,94,95]. Therefore, it is difficult to determine the compatibility of *F. graminearum* with wheat by outbreak of ROS in the infected position, but ROS produced in a pathogen-infected position may play a role in signaling pathways because ROS are closely related to the SA pathway.

## 5. Gene Expression Change Profiles Support the View That Photosynthesis Plays an Important Role in the Formation of Resistance

The changes in photosynthesis parameters, ROS bursts, and POD activity during compatibility and incompatibility responses can reveal only superficial changes in the plant-mediated immune defense response. The gene expression change profiles in the interaction between pathogens and host can systematically reveal the changes of physiological metabolism and signaling molecules involved in immune defense during the formation of host resistance [96,97,98]. During pathogen invasion, the changes of host photosynthesis were associated with the expression of photosynthesis-related genes [99]. The expression of genes related to the regulation of ROS and plant hormone synthesis and catabolism can indicate the changes of ROS and hormone levels in the host [100]. For instance, some studies have indicated that the expression of photosynthesis-related genes is generally downregulated under biotic stress, which is conducive to the production of ROS [101,102], while the upregulated expression of genes involved in the synthesis of JA, SA, and ethylene (ET) can stimulate the hormone-mediated defense response [103]. In addition, the accumulation of H_2_O_2_ in chloroplasts can lead to an increase in the level of SA [104], which in turn inhibits H_2_O_2_-scavenging enzymes such as CAT and APX [105]. In the process of interaction between host and pathogen, ROS, antioxidant enzymes, plant hormones, and photosynthesis constitute a complex mutual regulation network, and it is difficult to explore the relationship between them only by measuring physiological and biochemical indicators [106,107]. Therefore, comparing the changes in photosynthesis-related genes during the occurrence of different types of fungal diseases in wheat is beneficial for understanding the molecular mechanism through which wheat mediates different types of immune defense processes.

### 5.1. Global Downregulation of Photosynthesis-Related Genes in Response to Many Wheat Diseases May Disrupt Photosynthesis and Promote ROS Production

ROS, mainly H_2_O_2_, are key immune defense products of photosynthesis [84,108,109]. In incompatible reactions, the accumulation of ROS is usually accompanied by a decrease in the photosynthesis rate because the inhibition of photosynthesis is conducive to ROS production and accumulation [110,111]. Moreover, pathogens invasion usually results in the downregulation of photosynthesis-related genes in the host even if the host is not sensitive to the pathogen [65,112]. These findings indicate that active downregulation of photosynthesis-related genes in incompatible reactions can induce the accumulation of ROS, driven by a decrease in the photosynthesis rate of the host. This idea is supported by numerous reports of changes in the expression of photosynthesis-related genes caused by interactions between wheat and its pathogens [113,114,115]. For example, RNA sequencing of wheat infected by *Bgt* revealed that ROS bursts are accompanied by a global downregulation of photosynthesis-related genes, including those encoding chlorophyll a/b-binding (Cab) protein, ribulose bisphosphate carboxylase small chain (RbcS) protein, and ATP synthase, which parallel the decrease in the Pn [25]. Moreover, the significant decrease of chlorophyll fluorescence parameters (Fv’/Fm’, Fv/Fm, ΦPSII, ETR, qP) indicated that the photosynthetic electron transport chain of resistant wheat was blocked at the early stage of *Bgt* inoculation, which further supported that the decrease of photosynthesis was related to ROS production [25]. Transcriptome analysis of the susceptible cultivar Jingdong 8 and its resistant near-isogenic line also showed that the expression of a large number of photosynthesis-related proteins was inhibited at the early stage of inoculation with Bgt [116]. Additionally, the WKS1 protein encoded by stripe rust resistance gene *Yr36* can phosphorylate an extrinsic member of PSII and reduce photosynthetic rate [71]. In addition, comparison of the gene expression profiles of resistant wheat after inoculation with *Pst* revealed that the systems that produce ROS and nitric oxide (NO) are enriched, and the expression of photosynthesis-related genes is also reduced during this period [114]. Taken together, these results support the idea that active downregulation of photosynthesis-related genes disrupts photosynthesis as part of the incompatibility between parasitic fungi and wheat, which is associated with the activation of immune defenses.

Associations between *F. graminearum* infection and changes in photosynthesis in wheat have not been consistently recognized. Multiple gene expression profiles of FHB infection have suggested that the regulation of photosynthesis-related genes involving resistance to FHB is quite different from that involving stripe rust and powdery mildew. For example, analysis of the gene expression profile of QTL *FhbL693b*, which is a genomic region associated with FHB resistance, revealed that most of the differentially expressed genes detected in the region were related to photosynthesis, which indicates that photosynthesis may be involved in FHB resistance [74]. In addition, integrative transcriptome and hormone analysis indicated that SA and JA played a positive role in FHB resistance and that photosynthesis-related genes of the resistant variety Sumai 3 were downregulated at the early stage of inoculation [97]. Another study suggested that the ROS signaling pathways mediated by photosynthesis-related genes were not extremely important in FHB resistance because ROS-producing/scavenging systems were more active in the susceptible mutant than in the resistant variety Wangshuibai [94]. What is more, the transient susceptibility of the FHB-resistant line L693 and the inhibition of the ROS production system in Wangshuibai after inoculation indicated that photosynthesis-related genes did not directly induce the production of ROS to mitigate FHB resistance [74,94]. The development of resistance to FHB in wheat may depend on the activation of SAR by the SA and JA signaling pathways [97], which is also related to photosynthesis due to the synthesis of related hormones, mainly in chloroplasts [117].

### 5.2. Photosynthesis-Related Genes Directly Regulate the Immune Defense

In addition to producing ROS and regulating plant hormone levels to indirectly participate in plant immune defense, some proteins related to photosynthesis and genes related to photorespiration can directly regulate the defense response [118,119]. For example, the electron receptor protein ferredoxin-I (FD-I), which is involved in the photosynthetic process, is closely related to the production of ROS and the induction of the HR [120]. Inducing ferredoxin-like protein (AP1) expression in transgenic rice can increase resistance to *Xanthomonas oryzae* pv. *oryzae* [121]. In addition, compared with their wild-type counterparts, transgenic lines of *Oncidium* orchids are more resistant to soft rot disease when the coding sequence of ferredoxin-like protein (pflp) is transfected [122]. Moreover, transient overexpression of *glycolate oxidase* (*GOX2*), *serine glyoxylate aminotransferase* (*SGT*), and *serine hydroxyl methyltransferase* (*SHMT1*), which are involved in photorespiration in tobacco, increased the basic defense of transgenic lines against *P. syringae* [118]. On the other hand, interactions between pathogens and hosts revealed that many fungal effectors directly acted on chloroplasts and inhibited the immune response of plants by affecting the function of chloroplasts so that pathogens could successfully invade the host [20,123,124]. These clues suggest that several resistance genes may function together with photosynthesis to resist pathogen invasion.

Changes in the expression of photosynthesis-related genes in wheat during pathogen invasion and photosynthetic changes caused by wheat resistance genes can help to compensate for the gaps in knowledge concerning the mechanisms underlying resistance genes in wheat. Lots of studies have indicated that the development of resistance through the action of several wheat resistance genes relies on photosynthesis and chloroplasts [125,126,127]. For instance, to improve wheat powdery mildew resistance, the transfection of several receptor protein kinase-encoding genes can improve resistance to powdery mildew by inducing ROS bursts [90,126,127]. A recent discovery showed that protein kinase receptors could phosphorylate NADPH oxidase to activate ROS in *Arabidopsis thaliana*, which implies that there is a link between resistance genes of receptor protein kinase and photosynthesis in wheat [126,128,129]. In addition, the wheat stripe rust resistance protein WKS1 directly phosphorylates thylakoid-associated ascorbate peroxidase (TAPX) in chloroplasts and decreases its peroxide-scavenging ability [130]. The WKS1 also acts directly in chloroplasts to participate in immune defense responses. Moreover, Li et al. [74] identified three photosynthesis-related genes involved in the development of FHB resistance via transcriptome analysis in the chromosomal region of the resistance QTL *FhbL693B* [74]. Together, these reports show that photosynthesis plays a key role in wheat resistance and that wheat resistance genes may regulate photosynthesis to mediate resistance against pathogen invasion.

## 6. Summary and Future Efforts

**Figure 1:**
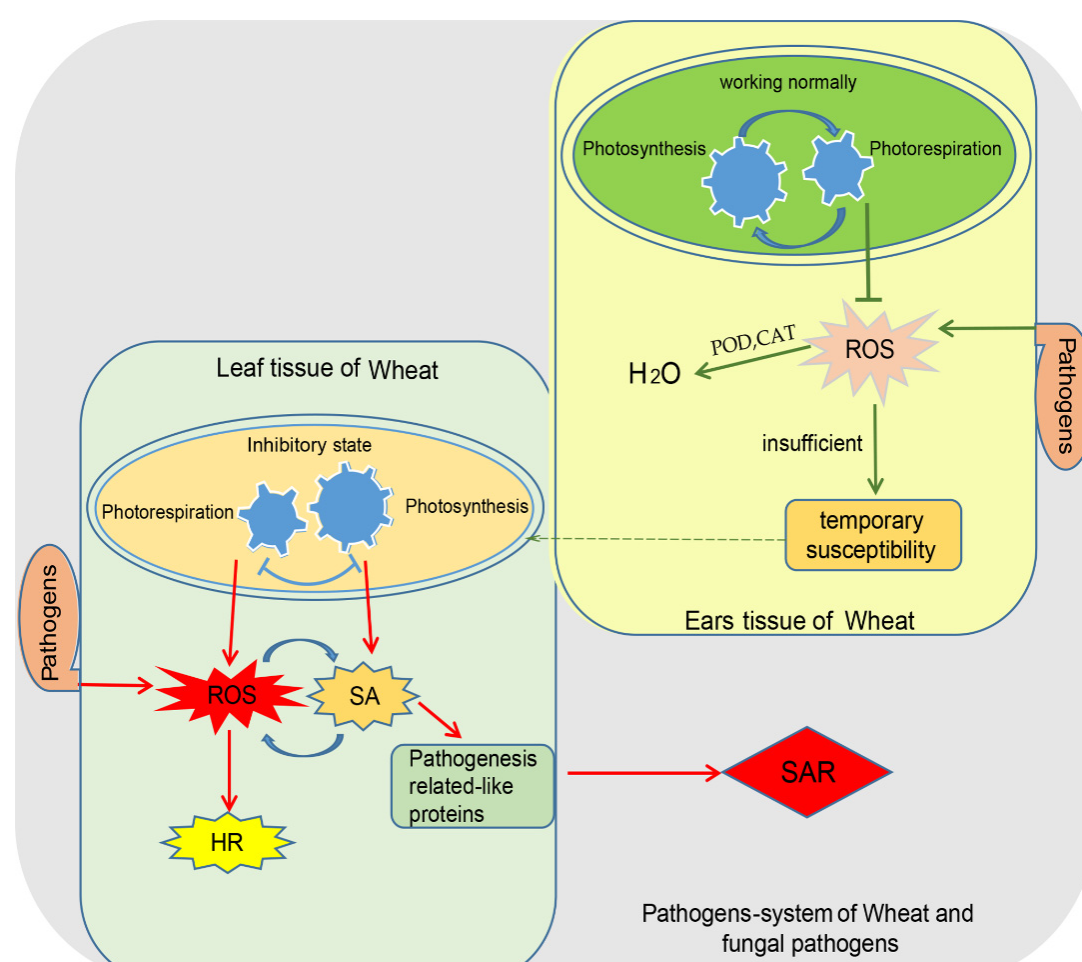
Hypothesized model of photosynthesis involving information concerning resistance to wheat source and sink diseases in the early stages of pathogen invasion.

Similar to how temperature can be an indicator of disease for a doctor, photosynthesis could be an important indicator for a plant pathologist speculating about the results of interactions between hosts and pathogens. Changes in photosynthesis parameters in the early stage (usually within 24 h) after pathogen infection could be an important causal agent contributing to the final resistance ability of plants, while changes in the late stage (usually 72 h) after pathogen infection could be the result of the resistance response. In addition, wheat and its main fungal pathogens, including *Bgt*, *Pst*, and *F. graminearum*, constitute ideal plant–pathogen systems because of their different infection positions from a photosynthetic standpoint. By providing an overview of the relevant data, we aimed to develop a model to explain the putative mechanism through which photosynthesis is involved in the wheat resistance response (Figure 1). Infection with source pathogens could cause a decrease in the photosynthesis rate, while infection with sink pathogens could lead to an increase in the photosynthesis rate at local infection positions, but both infection types directly and indirectly result in a decrease in the photosynthesis rate in source organs, which may be associated with SAR. Usually, the larger the decreased amplitude is, the greater the tendency of incompatibility. This could be explained in view of the accumulation of ROS, especially H_2_O_2_, in source organs, which could be further used as signaling molecules to activate the HR and SAR. In our opinion, a clearer relationship or defined role of photosynthesis in interactions between hosts and pathogens could be determined by additional solid evidence in the future.

## Figures and Tables

**Table 1:** Characteristics of the three major fungal diseases of wheat.

Major Diseases in Wheat	Pathogen	Position of Infection	Infection Period	Optimal Temperature	Classification of Diseases
Powdery mildew	*Blumeria graminis* f. sp. *tritici* (*Bgt*)	leaf	whole growth period	15–20 °C [32]	source disease
Stripe rust	*Puccinia striiformis* f. sp. *tritici* (*Pst*)	leaf	whole growth period	13–16 °C [36,37]	source disease
Fusarium head blight	*Fusarium graminearum* species complex	ear	adult stage	20–25 °C [39]	sink disease

**Table 2:** Changes in photosynthesis and antioxidant enzyme activity in response to the three main fungal pathogens in wheat at the early stage of infection (within 72 h).

Major Diseases in Wheat	Parameter (Leaf)	Wheat Cultivar/Line	Resistance	0 h	12 h	24 h	48 h	72 h
Powdery mildew	Pn [25]	L658	R	CG	↘	↗	→	↘
		L958	S	CG	↘	↗	→	↘
	SOD [25]	L658	R	CG	→	→	→	↘
		L958	S	CG	↗	→	↘	↘
	CAT [25]	L658	R	CG	↘	↗	↘	↗
		L958	S	CG	→	↗	↘	↘
Stripe rust	Pn [24]	CN19	R	CG	─	─	─	↘
		Sy95-71	S	CG	─	─	─	↘
	Pn [73]	*psbo-A1* Mutant	R	─	─	─	↘	─
		Kronos	S	─	─	─	CG	─
Fusarium head blight	Pn [74]	L693	R	CG	─	↘	→	→
		L661	S	CG	─	→	→	→
	SOD [74]	L693	R	CG	─	↘	→	→
		L661	S	CG	─	↗	→	→
	CAT [74]	L693	R	CG	─	↗	→	→
		L661	S	CG	─	↘	↘	↘

CP: control group; ↘: indicates that the current time has decreased significantly from the previous point in time; ↗: indicates that current time has increased significantly from the previous point in time; →: indicates that current time had no significant change from the previous point in time; ─: indicates that no available data were detected.

## Data Availability

Not applicable.
